# Rethinking tube feeding in palliative care: Impact on pneumonia, depression, and mortality in patients with dysphagia and life-limiting illness

**DOI:** 10.1371/journal.pone.0333895

**Published:** 2025-10-07

**Authors:** Jennifer Hanners Gutierrez, Kelly Klein, Milan Bimali, Jessica Sanders

**Affiliations:** 1 Department of Otolaryngology, School of Medicine, Texas Tech University Health Sciences Center, Lubbock, Texas, United States of America; 2 University Medical Center (UMC) Health System, Lubbock, Texas, United States of America; 3 Department of Family Medicine, Texas Tech University Health Sciences Center, Lubbock, Texas, United States of America; 4 Department of Biostatistics, University of Arkansas for Medical Sciences, Little Rock, Arkansas, United States of America; 5 Clinical Research Institute, Texas Tech University Health Sciences Center, Lubbock, Texas, United States of America; Neighborhood Physical Therapy, UNITED STATES OF AMERICA

## Abstract

**Introduction:**

Tube feeding continues to be recommended for nutrition when a patient has dysphagia amid life-limiting illness. Tube feeding is frequently claimed to be a safer alternative to oral feeding. Oral feeding, including careful hand feeding, has emerged as a viable and evidence-based alternative for patients who desire nutrition at the end of life but have trouble swallowing. There is a critical need to determine the best option for a feeding route at the end of life. This novel, pilot, prospective study was conducted to contribute toward that need. This is the first study of its kind.

**Aims:**

Researchers aimed to investigate the impact of feeding route (oral or tube) on patient outcomes, contributing to scientific knowledge as related to nutritional decision-making when patients are critically or terminally ill, desiring nutrition, and diagnosed with dysphagia.

**Methods:**

Participants (N = 65) were admitted to a tertiary center, diagnosed with dysphagia and life-limiting illness, and treated by Palliative or Family Medicine. Data were collected as related to demographic (age, sex, race, ethnicity) and clinical statistics (mortality risk/severity of illness, primary diagnoses, patient outcomes [pneumonia, depression, and mortality], and feeding route [oral and tube]). Logistic regression modeling (unadjusted and adjusted by mortality risk and age) and propensity score matching were used to analyze data.

**Results:**

Results indicated a greater likelihood of negative clinical outcomes as related to tube versus oral feeding. Findings challenge default use of tube feeding as a safer alternative for nutrition. Specifically, results revealed tube feeding to be significantly (p < 0.001) associated with increased risk of pneumonia (adjusted OR = 19.28, p < 0.01) and significantly (p < 0.001) associated with depression (adjusted OR = 17.25, p < 0.01), with mortality also trending higher (adjusted OR = 2.78, p = 0.147). Moreover, a composite outcome analysis (pneumonia, depression, or mortality) revealed impressively greater odds of an adverse event occurring due to tube versus oral feeding (adjusted OR = 55.64, p < 0.01), underscoring the need to consider further research and a possible paradigm shift in nutritional decision-making for palliative care patients. The propensity score analysis yielded balanced, matched groups (n = 30), 15 participants who were oral-fed and 15 who were tube-fed. Results also revealed significantly higher rates of pneumonia (p < 0.001) and depression (p = 0.035) in participants who received tube versus oral feeding. Again, mortality trended higher but significant results were not achieved.

**Clinical implications:**

These results have substantial clinical implications. Results support reconsidering routine tube feeding in favor of individualized, patient-centered approaches that prioritize quality of life and informed decision-making. This pilot study contributes to ongoing discussions about evidence-based palliative care and has the potential to influence guidelines on feeding practices for terminally-ill patients.

## Introduction

Patients with serious health care complications and life-limiting illness often experience difficulty swallowing (dysphagia) [1]. Difficulty swallowing manifests in different ways. During oral feeding, patients with dysphagia often struggle to transport the food from the anterior to posterior oral cavity, exhibit turbulent breathing after swallowing, require multiple swallows to propel the food through the pharynx, and/or robustly cough during eating or drinking. Dysphagia may lead to health care providers advocating for enteral (tube feeding) due to feared consequences of aspiration [2]. With this pilot study, researchers aimed to determine the best option for nutritional provision (oral or tube feeding) when a patient has a critical or terminal illness and dysphagia. This is important, because patients continue to be advised to choose a feeding tube over oral nutrition when they exhibit swallowing difficulty, often counseled that a tube is “safer.” Existing research does not support this practice. It is essential that health care providers be armed with data that may better inform nutritional decision-making at the end of life.

Pneumonia can be a consequence of aspiration (i.e., foreign material, such as oropharyngeal pathogens carried by food or drink, bypasses the vocal folds and enters the lungs). Past studies report pulmonary decompensation amid pneumonia due to oral feeding with dysphagia and aspiration. Evidence has also revealed harm from tube feeding in certain patient populations. Research has revealed pneumonia and mortality to increase when patients with advanced dementia [3,4], end-stage stroke, or end-stage Chronic Obstructive Pulmonary Disease (COPD) are fed via a tube versus by mouth. Depression has also been found to worsen with tube versus oral feeding [5]. Yet, the practice of recommending tube feeding persistently during terminal illness continues, even in the context of patients pleading to eat favorite foods by mouth at the end of life.

It should not be surprising that depression has been found to have a strong relationship to tube feeding [5,6]. When oral nutrition is withdrawn from a patient who is facing the end of life, past studies report increased isolation/lack of socialization and a negative impact on the sense of belonging [7]. Preferred foods and drinks are associated with positive relationships and memories which can fade amid cessation of oral nutrition [8]. Dignity and patient empowerment are also negatively affected by the loss of normalcy that aligns with tube feeding. Ironically, rhetoric used to convince patients that tube feeding is safer is misleading, as mortality has been repeatedly shown to be strongly associated with tube feeding [9–12].

The practice of health care providers advising tube feeding as a lower-risk alternative to oral feeding at the end of life is not supported by evidence and may work against principles of biomedical ethics: (a) respect for patient autonomy, (b) beneficence, (c) non-maleficence, and (d) justice [13]. *Patient autonomy* is supported when health care providers listen to the patient and strive to meet the patient’s expressed desires. This ethical principle is best achieved when the patient is educated on the risks and benefits of different feeding routes (oral versus tube); therefore, the patient, Medical Power of Attorney (MPOA), or alternative/surrogate decision maker can make an informed autonomous choice. Informed autonomous consent reflects patient or MPOA choice that is intentional, volunteered (non-coerced), based on a clear understanding of risks and benefits, and made by someone with capacity and authority to choose [14,15]. Informed consent requirements also include distinct components related to disclosure and understanding and are met if: (a) the health care provider takes care to fully disclose that which the patient may not understand (e.g., “If you choose this, here is what can happen”) and (b) the health care provider verifies patient or MPOA comprehensive understanding of education provided [16].

*Beneficence* means “to do good,” and *non-maleficence* means “do no harm.” While medical practitioners intend to make decisions that are in the best interest of their patients, the long-term implications may not be weighed or carefully considered. For example, family members, MPOAs, or alternative/surrogate decision makers may communicate concern over their loved one not eating in the context of advanced-stage dementia. In response to this concern, a physician may recommend initiation of tube feeding. Tube feeding has not been shown to improve longevity in this patient population. In contrast, it causes harm. The American Geriatrics Society [17] does not recommend tube feeding amid advanced dementia, but rather, careful hand oral feeding. *Justice* is the practice of ensuring decisions made across patients are fair and equitable. For example, careful hand oral feeding cannot be solely offered to patients with a strong social network and much support. Although sometimes daunting due to staffing shortages and administrative barriers, patients with little support must be offered the opportunity to receive assisted oral feeding as tolerated, because evidence supports this practice [18].

Palliative Medicine domains of practice must also be strongly considered when care is provided to patients with life-limiting diagnoses. Disease management and physical care are only part of what a provider must address when treating a patient at the end of life. Standards of care in Palliative Medicine also involve psychological, spiritual, and cultural support [19]. Reduction of suffering is key, as the World Health Organization [20] communicates this as the goal of palliative care. Tube feeding, per evidence, has been shown to cause psychosocial [21] and physiological distress as compared with oral feeding.

Outcome measures for this study (pneumonia, depression, mortality) were determined based on their likelihood of occurring in the context of dysphagia (inclusion criterion) and their association with the complexities of nutritional decision-making. Medical practitioners consistently strive to do the “right thing” when it comes to selecting a feeding route for patients continuing to desire nutrition at the end of life. The “right thing,” however, can be challenging to identify with pressures amidst the health care community to overmedicalize and/or provide optimal nourishment even when patients are only craving taste/gustatory stimulation which can be satisfied with limited nutritional volume/intake. Considerations as related to feeding route at the end of life should be heavily weighed and their impact on pneumonia, depression, and mortality should be explored.

Prior studies that have compared oral and enteral (tube) nutrition at the end of life have done so while analyzing feeding route in association with specific diagnoses, such as dementia, stroke, or COPD. For this pilot study, researchers determined a need to investigate the impact of feeding route (independent variable) on outcome measures (pneumonia, depression, mortality) across an array of life-limiting diagnostic categories: (a) neurological, (b) respiratory, (c) trauma, (d) immunosuppressive/viral, (e) wound/infection, (f) cancer, (g) cardiac, (h) renal disease, and (i) liver disease. This is the first study that has compared the effects of oral versus tube feeding in patients diagnosed with dysphagia and terminal illness (i.e., across diagnoses).

There is a research gap and a dearth of prospective trials investigating patient outcomes as related to oral versus tube feeding. Cintra et al. [3] conducted a prospective observational study on persons with advanced-stage dementia and reported significantly greater harm as related to pneumonia (p = 0.006) and mortality (at 3 months, p = 0.004 and at 6 months, p = 0.012) amid tube feeding compared to oral feeding. Wu et al. [22] compared oral to enteral feeding amid postoperative pancreatic fistula and found that oral feeding was non-inferior to tube feeding. Oral feeding significantly decreased the length of the hospital stay (p = 0.035) and overall associated costs (p < 0.001). Tai et al. [23] reported poor tolerance of nasogastric (NG) tube feeding compared to oral feeding in the context of advanced cirrhosis. These past studies do not effectively inform the health care community of the best option for a feeding route when critical or life-limiting illness exists in the context of dysphagia.

It is well known that dysphagia is often a resulting complication of neural insult. Researchers reported that 53% of patients admitted to a tertiary care center exhibited dysphagia following stroke [24]. Won et al. [25] reported dysphagia in 13% of patients following a subdural hematoma (SDH). Moreover, El Halabi et al. [26] reported dysphagia for a wide range of neurogenic conditions, including but not limited to dementia, Myasthenia Gravis, neurosyphilis, encephalitis, and polymyositis/dermatomyositis. Dysphagia was reported in 50% of patients diagnosed with Parkinson’s Disease and progressed from 30–100% incidence in patients diagnosed with Amyotrophic Lateral Sclerosis.

Dysphagia is also commonly associated with severe respiratory conditions. In a prospective study conducted by Langmore et al. [27], aspiration was detected in 33% of patients diagnosed with acute respiratory failure and who required at least 48 hours of mechanical ventilation. Li et al. [28] conducted a systematic review and meta-analysis and revealed dysphagia present in 32.7% of patients diagnosed with COPD. In 2024, Freeman-Sanderson et al. [29] reported dysphagia in 29% of patients following trauma, such as spinal cord injury (SCI), with mechanical ventilation increasing the likelihood of dysphagia and only 18% of these patients returning to a regular, non-modified diet by hospital discharge.

Nustas et al. [30] reported presence of dysphagia in 4.2% (8,699 of 206,332) of persons diagnosed with Human Immunodeficiency Virus (HIV) and hospitalized; dysphagia increased length of stay, costs associated with hospital admission, and rate of readmission within a 30-day period. Printza et al. [31] reported a high incidence of dysphagia with patients diagnosed with Covid-19. Results of this research revealed 63% of patients diagnosed with Covid-19 and hospitalized in an intensive care unit were aspirating and unable to eat by mouth. In 2023, researchers conducted a retrospective observational analysis and 55 out of 101 sepsis survivors were identified as having dysphagia [32]. After the 7^th^ day, presence of dysphagia lowered the chance of a patient being discharged home and increased the likelihood of total dependency.

It has long been known that dysphagia is a complication of head and neck cancer. Kuhn et al. [33] wrote an expert consensus statement and reported dysphagia incidence at 28% upon presentation of head and neck cancer and in 50% of pharyngeal cancers [34]. Dysphagia is present in up to 75% of survivors of head and neck cancer [35]. Dysphagia following heart failure is a unique area of study, and researchers recently prospectively detected dysphagia in 23.4% of patients admitted to a hospital with cardiac complications. Nativ-Zeltzer et al. [36] identified kidney disease as an independent risk factor for pneumonia in 689 patients diagnosed with dysphagia by instrumental testing (i.e., Video Fluoroscopic Swallow Study [VFSS]). Lastly, researchers have determined that new onset, chronic dysphagia (lasting more than 90 days) is present in 30% of patients with advanced cirrhosis following endoscopic band ligation (EBL) [37].

There remains a critical need to determine the best and least risk feeding route when a patient has dysphagia, desires nutrition, and is suffering from a life-limiting diagnosis. Researchers investigated patient outcomes (pneumonia, depression, mortality) based on feeding route (oral versus tube) with covariates (mortality risk and age) isolated from the treatment effect in the primary statistical analysis and participants matched on mortality risk, age, and diagnosis in the secondary statistical analysis, propensity score matching. Based on limited prior research, it was hypothesized that greater odds of negative patient outcomes would be associated with tube feeding versus oral feeding.

Nutrition plays a role in the journey of each patient. Food has been shown to contribute to both the physiological and psychological comfort of patients, and the withdrawal of oral nutrition has been shown to have a negative effect. The researchers’ overarching aim was to complete a pilot study that may establish a foundation on which future scientists can build as related to the best option for a feeding route at the end of life, and this aim was achieved. The results of this study will contribute to nutritional decision-making in Palliative Medicine.

## Methods

### Participants

Participants were patients admitted to a tertiary care center. The recruitment period for participants was August 20, 2020 to August 19, 2024. Participants were treated for serious health care complications by the Palliative Medicine team and/or Family Medicine. Data on outcome measures (pneumonia, depression, and mortality) and feeding route (oral versus tube) were collected. Data were also collected as related to covariates: (a) mortality risk and (b) age. Moreover, descriptive and clinical data were collected as related to participants’ primary diagnoses, severity of illness, sex, race, and ethnicity. Diagnostic categories proposed for inclusion in this study were as follows: (a) neurological, (b) respiratory, (c) trauma, (d) immunosuppressive/viral, (e) wound/infection, (f) cancer, (g) cardiac, (h) renal disease, and (i) liver disease. Diagnostic categories were chosen based on their frequent alignment with dysphagia, morbidity, and mortality. Data were collected prospectively through observation and electronic chart query in a single-center, using a convenience sampling approach with pre-specified eligibility criteria.

### Sample size and data collection

Researchers aimed to collect data over a 4-year period to provide an adequate sample size for statistical analysis. This study was not powered to detect a pre-specified effect size. The sample size estimation was driven by logistical and feasibility reasons. The study closest to the design of this research prospectively analyzed oral versus tube feeding amid advanced-stage dementia in 67 participants [3]. Participant consent was required. The Institutional Review Board (IRB) approved the study and associated consent forms. Written consent was obtained from each participant. The IRB mandated ethical rigor. All data were deidentified for the purpose of statistical analysis.

Researchers conducted electronic chart queries to collect data for this prospective observational analysis. The index disease (primary diagnosis) of each participant was determined based on the International Classification of Diseases (ICD)-10 code entered by the lead physician/health care provider. The outcome measure pneumonia was detected via ICD-10 coding if *newly diagnosed*. Physicians diagnosed pneumonia and entered this diagnosis by ICD-10 code based on radiology studies/chest imaging specific to pulmonary health, clinical symptoms, physical chest examinations, and laboratory markers/tests (e.g., sputum grain stain and culture, inflammatory markers such as procalcitonin, and blood cultures). The CURB (Confusion, Blood Urea Nitrogen [BUN], Respiratory Rate, Blood Pressure)-65 score was recorded during the participant’s hospital stay if pneumonia was *present on admission* (i.e., to determine worsening of pulmonary state or lack thereof, as related to feeding route). *Of note: the CURB-65 scores did not change throughout the hospital stay for the five participants diagnosed with pneumonia upon admission. The outcome measure depression was detected via ICD-10 coding if *newly diagnosed*. Physicians diagnosed depression and entered this diagnosis by ICD-10 code based on responses to the Patient Health Questionaire-9 (PHQ-9) and/or based on depression symptoms aligned with Diagnostic and Statistical Manual of Mental Disorders, Fifth Edition (DSM-5) criteria: (a) depressed mood or anhedonia (i.e., loss of interest); (b) appetite/weight changes; (c) sleep disturbances; (d) fatigue or loss of energy; (e) feelings of being unworthy or extreme guilt; (f) psychomotor agitation or retardation; (g) disrupted ability to think or concentrate, and (g) suicidal ideation. Likert scales were developed to assess degree of depression via self-report if *present on admission* (i.e., to determine worsening of depression or lack thereof, as related to feeding route). Likert scales were created by the researcher and approved by the IRB. In theory, the investigator would have asked each participant who qualified to rate depression pre- and post-hospital admission from 1–10 with 10 equal to, “My depression is the worst it has ever been.” No patient met the criteria to participate in the depression inventory. To clarify, Likert scales were authorized but not used. *Of note: for this particular study, investigators elected to use Likert scales versus the PHQ-9 if depression was coded by ICD-10 in the electronic medical record (EMR) as an *existing diagnosis*. This avoided emergent management of responses (by researchers) to the final question on the PHQ-9 related to suicidal ideation. The hospital organization has existing policies that require treatment protocols for thoughts of suicide, and the primary medical team is required to enact these protocols. The outcome measure mortality was detected via manual chart review and confirmed via the hospital’s Information Technology (IT) Department of Analytics.

The covariate mortality risk and corresponding descriptive data point severity of illness scores were determined by an algorithm informed by each participant’s health care complications and diagnoses and provided to researchers by IT Analytics. Age, sex, race, and ethnicity were obtained from participants’ patient profiles in their digital charts. The first author manually inspected each chart and spoke with each participant or authorized patient representative/Medical Power of Attorney (MPOA) after consent for research participation to determine the primary route of feeding (route of longest duration). The first author also conducted manual chart analysis for each participant to detect presence or absence of dysphagia, determined by: (a) health care provider diagnosis by ICD-10 code or documented in provider digital chart notes, (b) clinical examination of swallowing, or (c) instrumental examination of swallowing.

A total of 69 participants were consented for participation in this study. Due to Covid-19 consent protocols in 2020, MPOAs provided virtual informed consent for four participants amid visitation restrictions. The IRB later advised the authors that in-person consent was preferred due to the sensitive nature of the research topic and the terminal nature of the patient population. Therefore, researchers elected to withdraw four participants, yielding a final sample size of 65 participants in the analysis dataset.

Inclusion criteria were defined a priori as follows

- all adult ages (18 years of age or older);
- presence of dysphagia;
- presence of life-limiting illness/diagnosis;
- admitted to UMC and treated by Palliative Medicine and/or Family Medicine;
- patient or authorized representative chooses nutrition by informed autonomous consent, and
- mortality risk and severity of illness scores accessible via IT Analytics related to diagnoses found in the electronic medical record.

Participants who were actively dying (imminent death) or did not desire nor accept nutritional support were excluded from this research study. It was important to exclude patients who were actively dying and thereby not receiving nutrition, because inclusion of these participants would have negatively impacted generalizability of results.

### Design and statistical analysis

This study involved a prospective observational analysis of participants with life-limiting illness and dysphagia who had chosen either oral or enteral (tube) feeding for nutrition. Each participant received oral care four times per day, per the organization’s oral hygiene protocol. Participants who received tube feeding or participants who received oral feeding and had a history of gastrointestinal abnormalities, such as reflux, had positioning restrictions and were not reclined below 30 degrees per the organization’s protocols. The dependent variables were pneumonia, depression, and mortality. The independent variable was feeding route (oral or tube). Pneumonia was identified from ICD-10 scores or CURB-65 scores and defined as newly diagnosed/worsening during admission or absent. Depression was identified from ICD-10 scores or Likert self-report scales and defined as newly diagnosed/worsening during admission or absent. The outcome measure mortality was identified through manual chart review and confirmed through an electronic query. It was defined as discharge to hospice (i.e., prognosis of 6 months or less to live) or patient death.

Covariates were mortality risk and age. Mortality risk is defined as the “likelihood of dying” (p. 8) according to the Methodology Overview of the 3M^TM^ All Patient Refined Diagnosis Related Groups [38]. Age (dichotomous) was defined as years of living. Descriptive data point severity of illness is defined as “the extent of physiologic decompensation or organ system loss of function” (p. 8). Both mortality risk and severity of illness are derived from calculating the health impact of a patient’s medical diagnoses and health care complications, and they have been determined to be the most accurate comorbidity indices [39]. Mortality risk and severity of illness scores are defined as follows: (a) score of 1 = minor, (b) score of 2 = moderate, (c) score of 3 = major, and (d) score of 4 = extreme. The algorithm assigns metrics to each patient’s physiological stability or lack thereof (including and beyond the primary and secondary diagnoses). Resulting metrics are extrapolated from a vast array of health care complications, including but not limited to malnutrition, obesity, conditions resulting from hormonal imbalance, conditions resulting from metabolic disturbances, and need for surgical or invasive procedures [40].

Sex (dichotomous) was defined as biological identity. As related to descriptive clinical data, index diseases ultimately included in the study (assigned to diagnostic categories neurological, respiratory, trauma, immunosuppressive/viral, cancer, and renal disease) corresponded to an ICD-10 code and were defined as dichotomous (existing or absent). Race was defined as: (a) American Indian or Alaska native, (b) Asian, (c) Black or African American, (d) Native Hawaiian or Other Pacific Islander, or (e) White. Ethnicity was defined as: (a) Hispanic or Latino or (b) Not Hispanic or Latino.

The primary aim of this study was to evaluate the association between feeding route (oral versus tube) and clinical outcomes among patients who desire nutrition amid a life-limiting illness and dysphagia. The outcome measures were pneumonia, depression, and mortality. The secondary aim was to contribute to shifting the nutritional decision-making paradigm in Palliative Medicine.

Descriptive statistics were utilized for baseline demographic and clinical characteristics. Continuous variables were summarized as mean (standard deviation), median (interquartile range), and range (minimum-maximum), while categorical variables were presented as frequency (percentage). Differences in distribution of continuous variables across the two study arms were tested for statistical significance using the Wilcoxon rank-sum test. Bivariate association between study arms and categorical variables were tested for statistical significance using either the Chi-squared test or Fisher’s exact test.

The association between feeding routes and the three clinical outcomes was assessed separately using **logistic regression modeling**. Logistic regression was selected because it estimates the odds of an outcome while adjusting for potential confounders, allowing for direct assessment of the independent association between feeding route and clinical outcomes. Given the low number of events in the analysis dataset, we decided to limit the number of confounders to two to avoid model overparameterization [41]. Based on findings from the exploratory data analysis, there were significant differences in age, mortality risk scores, and illness severity scores between the two study groups. Additional analysis further revealed that mortality risk scores and illness severity scores were significantly correlated. Hence, we decided to include age and the mortality risk index as confounders in the logistic regression model. Logistic regression modeling was done sequentially as follows: 1) first, an unadjusted analysis was completed with the feeding route as the independent variable; 2) secondly, an adjusted analysis was done by including age and mortality risk scores into the model from step 1.

We conducted an additional statistical analysis with propensity matched data to obtain two comparable groups to assess the effect of feeding route on clinical outcomes. While logistic regression adjusts for measured confounders within the regression model, **propensity score matching** creates a pseudo-randomized dataset by balancing baseline characteristics between groups prior to outcome assessment. Patients who were tube-fed were matched to patients who were oral-fed using cardinality matching [42]. The matched dataset had 30 patients (15 in tube-fed group and 15 in oral-fed group). Matching was employed to optimize comparability between groups, yielding a matched dataset that achieved balance not only in terms of matching variables (mortality risk, age, and diagnosis), but also with respect to sex, race, ethnicity, and severity of illness.

A two-sided p-value of 0.05 was used to determine statistical significance. The uncertainty in parameter estimates was reported using a two-sided 95% confidence interval. The analysis was done using R version 4.4.1.

## Results

This prospective study examined the association between feeding route (oral versus tube) and clinical outcomes (pneumonia, depression, and mortality) in patients with dysphagia and life-limiting illnesses. The analysis included 65 participants, with 41 receiving oral feeding and 24 receiving tube feeding. Overall, results revealed stronger associations with tube feeding and adverse outcomes. Based on study findings, there is greater likelihood that pneumonia and depression will occur amid tube feeding versus oral feeding in patients with dysphagia who are critically or terminally ill. Mortality also trended upward in participants who were tube-fed versus oral-fed.

Baseline demographic and clinical characteristics are summarized (see Tables 1 and 2). The mean age of participants was 70.66 years (SD = 14.18), with patients in the oral feeding group being significantly older than those in the tube feeding group (p = 0.032). Males comprised 62% of the study population, with no significant difference between feeding groups (p = 0.14). The majority of participants were White (89%), with no significant racial or ethnic differences between groups. Clinically, patients receiving tube feeding had significantly higher mortality risk (p < 0.001) and illness severity (p < 0.001) compared to those receiving oral feeding. *Importantly, mortality risk was adjusted for in the primary statistical analyses to ensure that the treatment effect was isolated from the potential confounding impact of health care status.

**Table 1:** Baseline Demographic Variables – Descriptive Statistics.

Characteristic	Overall, N = 65	Oral, N = 41	Tube, N = 24	P-value^1^
Age				0.032
N	65	41	24	
Mean (SD)	70.66 (14.18)	74.02 (10.90)	64.92 (17.27)	
Median (IQR)	72.00 (20.00)	75.00 (17.00)	65.00 (26.50)	
(Min., Max.)	(18.00,94.00)	(49.00,94.00)	(18.00,89.00)	
Sex				0.140
Male	40 (62%)	28 (68%)	12 (50%)	
Female	25 (38%)	13 (32%)	12 (50%)	
Race				0.800
Asian	2 (3.1%)	1 (2.4%)	1 (4.2%)	
African American	5 (7.7%)	4 (9.8%)	1 (4.2%)	
White	58 (89%)	36 (88%)	22 (92%)	
Ethnicity				0.800
Hispanic/Latino	23 (35%)	15 (37%)	8 (33%)	
Not Hispanic/Latino	42 (65%)	26 (63%)	16 (67%)	

1: P-values are based on Fisher’s exact test (when > 25% of expected cell counts are < 5) or Pearson’s Chi-squared test for categorical variables, and the Wilcoxon rank-sum test for continuous variables.

**Table 2:** Baseline Clinical Variables – Descriptive Statistics.

Characteristic	Overall, N = 65	Oral, N = 41	Tube, N = 24	P-value^1^
Diagnosis				0.400
Neurological	24 (37%)	17 (41%)	7 (29%)	
Respiratory	19 (29%)	9 (22%)	10 (42%)	
Trauma	5 (7.7%)	4 (9.8%)	1 (4.2%)	
Immunosuppressive	3 (4.6%)	1 (2.4%)	2 (8.3%)	
Cancer	11 (17%)	8 (20%)	3 (12%)	
Renal Disease	3 (4.6%)	2 (4.9%)	1 (4.2%)	
Unknown	0	0	0	
Mortality Risk				<0.001
Moderate	9/ 62 (15%)	9/ 38 (24%)	0 (0%)	
Major	22/ 62 (35%)	18/ 38 (47%)	4 (17%)	
Extreme	31/ 62 (50%)	11/ 38 (29%)	20 (83%)	
Unknown	3	3	0	
Illness Severity				<0.001
Moderate	3/ 62 (4.8%)	3/ 38 (7.9%)	0 (0%)	
Major	19/ 62 (31%)	18/ 38 (47%)	1 (4.2%)	
Extreme	40/ 62 (65%)	17/ 38 (45%)	23 (96%)	
Unknown	3	3	0	

n/ N (%).

1: P-values are based on Fisher’s exact test (when > 25% of expected cell counts are < 5) or Pearson’s Chi-squared test for categorical variables.

Participants were included if their primary diagnoses aligned with the following categories: neurological, respiratory, trauma, immunosuppressive/viral, cancer, or renal disease. For propensity score analysis, diagnoses were categorized as neurological, respiratory, and others (trauma, immunosuppressive/viral, cancer, or renal disease).

### Primary outcomes

The incidence of pneumonia was significantly higher in tube feeding group compared to the oral feeding group (79% vs 12%, p < 0.001) (see Table 3). Logistic regression analysis showed that tube feeding was associated with significantly increased odds of pneumonia (adjusted OR = 19.28, 95% CI: 4.5–109.6, p < 0.01), even after controlling for age and mortality risk (see Table 4). Depression was significantly higher among those receiving tube feeding (50% vs 9.8%, p < 0.001) compared to oral feeding. Adjusted logistic regression indicated a strong association between tube feeding and depression (adjusted OR = 17.25, 95% CI: 3.13–158.78, p < 0.01) (see Table 5). Mortality was observed in 18 participants (28%), with a higher death rate in the tube feeding group (46%) compared to the oral feeding group (17%) (p = 0.012). Although the unadjusted odds ratio suggested an increased risk of mortality with tube feeding (OR = 4.11, 95% CI: 1.34–13.47, p = 0.015), the adjusted model did not reach statistical significance (adjusted OR = 2.78, 95% CI: 0.71–11.7, p = 0.147) (see Table 6). The incidence of at least one adverse event (i.e., pneumonia, depression, or mortality) was significantly higher among participants receiving tube feeding (96% vs 37%, p < 0.001) (see Table 3).

**Table 3:** Clinical Outcomes – Descriptive Statistics.

Characteristic	Overall, N = 65	Oral, N = 41	Tube, N = 24	P-value
Pneumonia				<0.001
0: No	41/ 65 (63%)	36/ 41 (88%)	5/ 24 (21%)	
1: Yes	24/ 65 (37%)	5/ 41 (12%)	19/ 24 (79%)	
Unknown	0	0	0	
Depression				<0.001
0: No	49/ 65 (75%)	37/ 41 (90%)	12/ 24 (50%)	
1: Yes	16/ 65 (25%)	4/ 41 (9.8%)	12/ 24 (50%)	
Unknown	0	0	0	
Mortality				0.012
0: No	47/ 65 (72%)	34/ 41 (83%)	13/ 24 (54%)	
1: Yes	18/ 65 (28%)	7/ 41 (17%)	11/ 24 (46%)	
Unknown	0	0	0	
PDM				<0.001
0: No	27/ 65 (42%)	26/ 41 (63%)	1/ 24 (4.2%)	
1: Yes (at least one)	38/ 65 (58%)	15/ 41 (37%)	23/ 24 (96%)	
Unknown	0	0	0	

n/ N (%).

1: P-values are based on Fisher’s exact test (when > 25% of expected cell counts are < 5) or Pearson’s Chi-squared test for categorical variables. Note: PDM means at least one of Pneumonia or Depression or Mortality was observed.

**Table 4:** Association between Pneumonia and Feeding Mechanism.

Characteristic	Unadjusted Odds Ratio (95% CI)	Adjusted^1^ Odds Ratio (95% CI)	Adjusted Probability of Pneumonia
Feeding Route: Tube	27.36 (7.68,119.81) P-value: < 0.010	19.28 (4.5,109.6) P-value: < 0.010	0.76 (0.51,0.91)
Feeding Route: Oral (Ref)	–	–	0.14 (0.06, 0.31)

1: Adjusted for Age and Mortality Risk score (Extreme vs Moderate/Major).

**Table 5:** Association between Depression and Feeding Mechanism.

Characteristic	Unadjusted Odds Ratio (95% CI)	Adjusted^1^ Odds Ratio (95% CI)	Adjusted Probability of Depression
Feeding Route: Tube	9.25 (2.68,38.36) P-value: < 0.010	17.25 (3.13,158.78) P-value: < 0.010	0.55 (0.30,0.78)
Feeding Route: Oral (Ref)	–	–	0.07 (0.02, 0.21)

1: Adjusted for Age and Mortality Risk score (Extreme vs Moderate/Major).

**Table 6:** Association between Mortality and Feeding Mechanism.

Characteristic	Unadjusted Odds Ratio (95% CI)	Adjusted^1^ Odds Ratio (95% CI)	Adjusted Probability of Mortality
Feeding Route: Tube	4.11 (1.34,13.47) P-value: 0.015	2.78 (0.71,11.7) P-value: 0.147	0.41 (0.21,0.65)
Feeding Route: Oral (Ref)	–	–	0.20 (0.10,0.37)

1: Adjusted for Age and Mortality Risk score (Extreme vs Moderate/Major).

A *composite outcome variable* was created to assess whether **any of the three conditions**—pneumonia, depression, or mortality (PDM)—were present. This composite variable was created to shift the lens and view participants’ physiological and psychological responses to feeding route holistically, recognizing that the occurrence of even one of these outcomes represents a meaningful decline in patient health and may warrant changes in clinical management. An adverse outcome was defined as increased odds of either pneumonia, depression, or mortality occurring based on feeding route (oral or tube). An adverse outcome (i.e., at least one outcome measure increasing as related to feeding route) was observed in 38 participants (58%), with a strikingly high prevalence in the tube feeding group (96%) compared to the oral feeding group (37%) (p < 0.001). Logistic regression analysis revealed that tube feeding was associated with a significantly increased risk of experiencing at least one adverse effect (adjusted OR = 55.64, 95% CI: 7.59–1235.15, p < 0.01), demonstrating a significant association between feeding method and negative clinical aftermaths (see Table 7).

**Table 7:** Association between PDM (at least one of Pneumonia/Depression/Mortality) and Feeding Mechanism.

Characteristic	Unadjusted Odds Ratio (95% CI)	Adjusted^1^ Odds Ratio (95% CI)	Adjusted Probability of PDM
Feeding Route: Tube	39.87 (7.26,749.47) P-value: < 0.010	55.64 (7.59,1235.15) P-value: < 0.010	0.97 (0.78,0.99)
Feeding Route: Oral (Ref)	–	–	0.35 (0.20,0.55)

1: Adjusted for Age and Mortality Risk score (Extreme vs Moderate/Major).

### Propensity-matched analysis

A propensity-matched dataset of 30 participants (15 oral, 15 tube) was analyzed (see Table 8). Results from propensity-matched data reinforced previous findings, showing significantly higher rates of pneumonia (73% vs 13%, p < 0.001) and depression (47% vs 6.7%, p = 0.035) in the tube feeding group (see Table 9). Mortality remained higher but was not statistically significant in the matched cohort (40% vs 27%, p = 0.4). The persistence of significant differences even after controlling for potential confounders further supports the observed trends and associations.

**Table 8:** Distribution of Baseline Variables in Propensity Matched Data.

Characteristic	Overall, N = 30	Oral, N = 15	Tube, N = 15	P-value^1^
Mortality Risk				>0.900
Moderate/Major	8 (27%)	4 (27%)	4 (27%)	
Extreme	22 (73%)	11 (73%)	11 (73%)	
Diagnosis				>0.900
Neurological	10 (33%)	5 (33%)	5 (33%)	
Respiratory	12 (40%)	6 (40%)	6 (40%)	
Others	8 (27%)	4 (27%)	4 (27%)	
Age				0.600
N	30	15	15	
Mean (SD)	69.00 (16.30)	69.27 (11.63)	68.73 (20.37)	
Median (IQR)	70.00 (21.50)	67.00 (18.00)	77.00 (21.50)	
(Min., Max.)	(18.00,89.00)	(49.00,86.00)	(18.00,89.00)	
Illness_Severity2				0.600
Moderate/Major	4 (13%)	3 (20%)	1 (6.7%)	
Extreme	26 (87%)	12 (80%)	14 (93%)	
Sex				0.700
Male	17 (57%)	9 (60%)	8 (53%)	
Female	13 (43%)	6 (40%)	7 (47%)	
Race				0.200
Asian	1 (3.3%)	0 (0%)	1 (6.7%)	
African American	3 (10%)	3 (20%)	0 (0%)	
White	26 (87%)	12 (80%)	14 (93%)	
Ethnicity				0.400
Hispanic/Latino	10 (33%)	6 (40%)	4 (27%)	
Not Hispanic/Latino	20 (67%)	9 (60%)	11 (73%)	

n/ N (%).

1: P-values are based on Fisher’s exact test (when > 25% of expected cell counts are < 5) or Pearson’s Chi-squared test for categorical variables, and the Wilcoxon rank-sum test for continuous variables.

**Table 9:** Clinical Outcomes – Bivariate Association.

Characteristic	Overall, N = 30	Oral, N = 15	Tube, N = 15	P-value
Pneumonia				<0.001
No	17 (57%)	13 (87%)	4 (27%)	
Yes	13 (43%)	2 (13%)	11 (73%)	
Depression				0.035
No	22 (73%)	14 (93%)	8 (53%)	
Yes	8 (27%)	1 (6.7%)	7 (47%)	
Mortality				0.400
No	20 (67%)	11 (73%)	9 (60%)	
Yes	10 (33%)	4 (27%)	6 (40%)	
PDM				0.014
No	9 (30%)	8 (53%)	1 (6.7%)	
Yes	21 (70%)	7 (47%)	14 (93%)	

Note: PDM means at least one of pneumonia or depression or mortality was observed.

### Summary of key findings

This study provides compelling statistical evidence that tube feeding is associated with significantly higher risks of pneumonia and depression, with mortality also trending higher. The PDM analysis further reflects the increase in adverse outcomes associated with tube feeding. Findings show trends toward harm with tube feeding versus oral feeding in the context of serious illness and difficulty swallowing, supporting the need for further exploration of reported associations. Findings challenge the long-held belief that tube feeding is a safer alternative, suggesting instead that it may contribute to greater clinical deterioration in patients with dysphagia and life-limiting illnesses. A paradigm shift in medical decision-making regarding feeding routes in palliative care may need to be considered, especially if continued research with a larger sample size and multi-site approach replicates results.

## Discussion

This pilot prospective study contributes to existing literature on the relationship of oral or tube feeding amid life-limiting illness and dysphagia. The critical finding is that pneumonia and depression were significantly higher in participants who were tube-fed versus oral-fed, and this finding supports the hypothesis. Participants who received tube feeding had 19.28 times the odds of developing pneumonia compared to those receiving oral feeding and 17.25 times the odds of developing depression compared to participants who were oral-fed. Mortality risk consistently trended upward with each statistical analysis but did not reach statistical significance. When a composite outcome variable was created (PDM) to analyze the relationship of either pneumonia, depression, or mortality to feeding route (oral or tube), strong statistical significance was attained. Participants who were tube-fed had 55.64 times more likelihood to develop an adverse outcome as compared to participants who were orally fed. These results were obtained via rigorous statistical analyses that included logistic regression modeling with the covariates mortality risk and age isolated from the treatment effect and propensity score matching with participants matched on mortality risk (moderate/major or extreme), diagnosis (neurological, respiratory, and others [trauma, immunosuppressive/viral, cancer, or renal disease]), and age.

### Comparison to previous research

The findings align with previous studies that have reported increased harm from tube feeding as related to oral feeding in specific patient populations (e.g., advanced dementia, stroke, COPD). Cintra et al. [3] investigated tube versus oral feeding in the context of advanced-stage dementia and found that pneumonia and mortality increased amid tube feeding compared to oral feeding. Hanners Gutierrez et al. [5] reported pneumonia to be 10.14 times more likely to occur with tube versus oral feeding. Increased incidence of depression (2.79) and mortality (3.02) were also noted to be worse with tube feeding compared to oral feeding. In a content-rich review of ethical principles at the end of life, Akdeniz et al. [43] reported increased aspiration pneumonia and gastrointestinal complications with tube feeding. Johnston et al. [44] reported that 43% of patients who received a percutaneous endoscopic gastrostomy (PEG) tube placement died within 1 week and 70% of those patients died due to respiratory disease. In contrast, careful hand oral feeding as an alternative to tube feeding has been found to improve both psychosocial and physiological aspects of nutrition.

### Clinical implications, strengths, and limitations

The significant association between tube feeding and adverse outcomes reinforces the need for an improved approach to nutritional decision-making in palliative care. Evidence supports prioritization of patient autonomy and quality of life when discussing feeding options with patients who have dysphagia and are pleading to eat by mouth. The results of this groundbreaking pilot study reveal that oral feeding or careful hand oral feeding may be a viable and less harmful alternative to tube feeding for patients with dysphagia and life-limiting illness. Past studies have revealed the need for greater emotional support as dependence for cares increases [45], and interactive, careful hand feeding offers one way to provide this type of support [46]. With 55.64 greater odds of developing at least one adverse event (pneumonia, depression, mortality) due to tube feeding as compared to oral feeding, health care providers must heavily weigh the choice to feed someone enterally (by tube). Tube feeding should not be implemented by default for patients exhibiting swallowing problems when they are terminally ill.

This study has several notable strengths. The researchers completed a pilot study to investigate a key element of end-of-life care which required fortitude necessary to consent participants while they were enduring serious emotional and physical challenges. Several participants felt that they could leave a legacy through their participation. Offering this type of psychological support and establishing a connection with participants in this manner was and is indeed a strength. This is the first study to compare the impact of oral or tube feeding on pneumonia, depression, and mortality across multiple life-limiting diagnoses. The prospective nature of the study supported systematic data collection. Moreover, the use of logistic regression and propensity score matching enhanced the robustness of the findings and isolated potential confounding variables, such as age and mortality risk, from the treatment effect. Strong statistical significance was achieved across analyses, raising confidence in results. Lastly, the ICC values (1.0) indicated excellent interrater and intrarater agreement, demonstrating excellent consistency in data collection. These results enhance the credibility of the findings.

There are limitations that must be acknowledged. This pilot, exploratory study had a relatively small sample size (N = 65). However, the sample size aligns with the only other known prospective study to compare oral and tube feeding in patients with dysphagia at the end of life (N = 67) [3]; thus, contributing to a meaningful body of evidence and providing a foundation for future research. Additionally, the study was conducted in a single tertiary care center. These limitations present an opportunity for similar research to be conducted on a broader scale across multiple institutions, increasing the sample size and diversifying the settings. Expanding the scope of future studies may replicate these findings and enhance generalizability.

### Future research and conclusion

Further research is needed to explore the impact of feeding route (oral or tube) on outcomes (pneumonia, depression, mortality) when a patient desires nutrition amid a life-limiting illness and dysphagia. Larger-scale studies should aim to replicate these findings in diverse patient populations and investigate additional factors such as caregiver burden. Additionally, qualitative research exploring patient and family perspectives on nutritional decision-making could provide valuable insights into the psychosocial aspects of nutritional management at the end of life. It would be fruitful to explore protocols and perspectives of health care providers as related to understanding and disclosure of risks and benefits of different feeding routes and compare the perspectives of patients, MPOAs, or authorized patient representatives.

The results of this study provide compelling evidence that tube feeding in patients with dysphagia and life-limiting illness is associated with increased risks of pneumonia and depression, with a strong trend toward higher mortality. These findings challenge traditional assumptions regarding the safety and efficacy of tube feeding in palliative care and highlight the need for an evidence-based change in clinical practice. Findings support increased educational efforts to ensure health care providers are armed with well-grounded and recent evidence related to selection of a feeding route in the context of serious or terminal illness. An infrastructural health care shift toward a standardized feeding route selection protocol may be fruitful to ensure best practices are enacted (e.g., adherence to biomedical ethical principles, informed autonomous consent) versus default use or overuse of tube feeding in the context of dysphagia, and legislation should be proposed to solidify educational and clinical paradigm changes. Future research should build upon this foundation to refine evidence-based guidelines that prioritize biomedical ethics, psychosocial health/support, physiological comfort, and reduction of suffering in end-of-life nutrition decision-making.
